# Intravesical Bacillus Calmette–Guérin Treatment for T1 High-Grade Non-Muscle Invasive Bladder Cancer with Divergent Differentiation or Variant Morphologies

**DOI:** 10.3390/cancers13112615

**Published:** 2021-05-26

**Authors:** Makito Miyake, Nobutaka Nishimura, Kota Iida, Tomomi Fujii, Ryoma Nishikawa, Shogo Teraoka, Atsushi Takenaka, Hiroshi Kikuchi, Takashige Abe, Nobuo Shinohara, Eijiro Okajima, Takuto Shimizu, Shunta Hori, Norihiko Tsuchiya, Takuya Owari, Yasukiyo Murakami, Rikiya Taoka, Takashi Kobayashi, Takahiro Kojima, Naotaka Nishiyama, Hiroshi Kitamura, Hiroyuki Nishiyama, Kiyohide Fujimoto

**Affiliations:** 1 Department of Urology, Nara Medical University, Kashihara, Nara 634-8522, Japan; ffxxxx.nqou@gmail.com (N.N.); kota1006ida@yahoo.co.jp (K.I.); horimaus@gmail.com (S.H.); tintherye@gmail.com (T.O.); kiyokun@naramed-u.ac.jp (K.F.); 2 Department of Diagnostic Pathology, Nara Medical University, Kashihara, Nara 634-8522, Japan; fujiit@naramed-u.ac.jp; 3 Department of Urology, Faculty of Medicine, Tottori University, Yonago, Tottori 683-8503, Japan; ryoma_nishikawa@yahoo.co.jp (R.N.); teraoka-1119@tottori-u.ac.jp (S.T.); atake@med.tottori-u.ac.jp (A.T.); 4 Department of Urology, Graduate School of Medicine, Hokkaido University, Sapporo, Hokkaido 060-8638, Japan; hiroshikikuchi16@yahoo.co.jp (H.K.); takataka@rf6.so-net.ne.jp (T.A.); nozomis@mbj.nifty.com (N.S.); 5 Department of Urology, Nara City Hospital, Nara 630-8305, Japan; jiro_eddie@yahoo.co.jp; 6 Department of Urology, Saiseikai Chuwa Hospital, Nara 633-0054, Japan; takutea19@gmail.com; 7 Department of Urology, Faculty of Medicine, Yamagata University, Yamagata 990-9585, Japan; ntsuchiya@med.id.yamagata-u.ac.jp; 8 Department of Urology, School of Medicine, Kitasato University, Sagamihara, Kanagawa 252-0374, Japan; yaskiyomura@yahoo.co.jp; 9 Department of Urology, Faculty of Medicine, Kagawa University, Kagawa 761-0793, Japan; rikiya@med.kagawa-u.ac.jp; 10 Department of Urology, Graduate School of Medicine, Kyoto University, Kyoto 606-8507, Japan; selecao@kuhp.kyoto-u.ac.jp; 11 Department of Urology, Faculty of Medicine, University of Tsukuba, Tsukuba, Ibaraki 305-8576, Japan; tkojima@md.tsukuba.ac.jp (T.K.); nishiuro@md.tsukuba.ac.jp (H.N.); 12 Department of Urology, Faculty of Medicine, University of Toyama, Toyama 633-0054, Japan; nishiyan@med.u-toyama.ac.jp (N.N.); hkitamur@med.u-toyama.ac.jp (H.K.)

**Keywords:** urinary bladder neoplasms, Bacillus Calmette–Guérin (BCG), immunotherapy, divergent differentiation, variant morphology, survival

## Abstract

**Simple Summary:**

The 2016 World Health Organization classification system distinguishes between urothelial carcinomas (UCs) with divergent differentiation (DD) and those with variant morphologies (VMs), which until now had been considered to indicate highest-risk cases. Intravesical Bacillus Calmette–Guérin (BCG) treatment is an alternative therapeutic and adjuvant option after transurethral resection of a bladder tumor. However, data comparing oncological outcomes after intravesical BCG treatment among pure UC, UC with DD, and UC with VMs are sparse. This is a retrospective study to investigate the outcomes of bladder-preservation therapy using intravesical BCG treatment on cases of bladder UCs with DD or VMs. We followed the outcomes of 1490 patients with pure UCs, UC with DD, or UC with VM. We found that concomitant VMs, not DD, was more likely to result in cancer-specific death. The VM-associated risk was significant for cancer-specific mortality only, not for recurrence-free or progression-free survival rates.

**Abstract:**

The 2016 World Health Organization classification newly described infiltrating urothelial carcinoma (UC) with divergent differentiation (DD) or variant morphologies (VMs). Data comparing oncological outcomes after bladder-preservation therapy using intravesical Bacillus Calmette–Guérin (BCG) treatment among T1 bladder pure UC (pUC), UC with DD (UC-DD), and UC with VMs (UC-VM) are limited. We evaluated 1490 patients with T1 high-grade bladder UC who received intravesical BCG during 2000–2019. They were classified into three groups: 93.6% with pUC, 4.4% with UC-DD, and 2.0% with UC-VM. Recurrence-free, progression-free, and cancer-specific survival following intravesical BCG were compared among the groups using multivariate Cox regression analysis, also used to estimate inverse probability of treatment weighting-adjusted hazard ratio and 95% confidence interval for the outcomes. Glandular differentiation and micropapillary variant were the most common forms in the UC-DD and UC-VM groups, respectively. Of 1490 patients, 31% and 13% experienced recurrence and progression, respectively, and 5.0% died of bladder cancer. Survival analyses revealed the impact of concomitant VMs was significant for cancer-specific survival, but not recurrence-free and progression-free survival compared with that of pUC. Our analysis clearly demonstrated that concomitant VMs were associated with aggressive behavior in contrast to concomitant DD in patients treated with intravesical BCG.

## 1. Introduction

Bladder cancer is the 10th most common cancer, with roughly 549,000 initially diagnosed cases and 200,000 deaths reported annually according to 2018 worldwide cancer statistics [1]. Non-muscle-invasive bladder cancer (NMIBC; Ta, T1, and Tis) is a heterogeneous disease accounting for approximately 70% of initially diagnosed bladder cancers [2]. Although most of NMIBC can be treated with the combination of transurethral resection of a bladder tumor (TURBT) and intravesical treatment of chemotherapy and Bacillus Calmette–Guérin (BCG), some patients have repeated intravesical recurrence and disease progression.

The European Association of Urology guidelines 2019 specify characteristics of the highest-risk subset from high-risk non-muscle invasive bladder cancers (NMIBCs) [3]. Patients are stratified into highest-risk NMIBC when the tumor has at least one of the following five aggressive factors: i) T1/high-grade (HG) urothelial carcinoma (UC) with concomitant bladder carcinoma in situ (CIS); ii) multiple and/or large and/or recurrent T1/HG UC; iii) T1/HG UC with prostate-involving CIS; iv) lymphovascular invasion (LVI); v) histological variants of UC; or vi) BCG-unresponsive NMIBC. Although immediate radical cystectomy (RC) should be considered for highest-risk NMIBC, this highly invasive intervention may be associated with a significant risk of overtreatment. Clinical practice guidelines suggest that intravesical BCG treatment is still an alternative therapeutic and adjuvant option after transurethral resection of a bladder tumor (TURBT) for high-/highest-risk NMIBCs [4,5,6]. Clinicians may judge that not all the patients with highest-risk NMIBC require RC because of the highly heterogenic nature of highest-risk NMIBC [7].

The 2016 World Health Organization (WHO) classification of tumors of the urothelial tract newly described or better defined ‘infiltrating UC with divergent differentiation (DD)’ and ‘infiltrating UC with variant morphologies (VMs),’ implying that the two subsets should be labeled separately to optimize the therapeutic strategy [8]. The former includes squamous, glandular, and trophoblastic differentiation, whereas the latter includes nested, microcystic, micropapillary, lymphoepithelioma-like, plasmacytoid/signet ring cell/diffuse, sarcomatoid, giant cell, poorly differentiated, lipid-rich, and clear cell (glycogen-rich) variants. These unusual types of UC have been called ‘histological variants’ and clubbed together, which is one of the inclusion characteristics of highest-risk NMIBCs. Recent studies evaluated the frequency of DD and VMs in invasive UC, reporting that squamous differentiation was the most common in DD and micropapillary variant was the most common in VMs, respectively [9,10]. NMIBCs, MIBCs, and metastatic diseases with DDs and VMs respond poorly to intensive surgery with chemotherapy or radiotherapy, resulting in worse survival outcomes [11,12,13,14,15]. As some VMs are extremely rare, small sample size hinders appropriate evaluation of their clinical relevance, especially in response to intravesical BCG treatment [16,17,18].

Squamous and glandular differentiation is seen at a relatively high frequency among histological variants. However, there are only a few small studies or case reports regarding the efficacy of intravesical BCG against NMIBC with VMs [16,19,20,21]. There are only sparse data comparing oncological outcomes after intravesical BCG treatment among pure UC (without any histological variant), UC with DD, and UC with VMs considering the newly adopted WHO 2016 classification system. Given the little information regarding the efficacy of BCG in UC variants, this study aimed to shed more clarity on the mortality and survival outcomes by retrospectively examining the outcomes of 1490 patients with UC who were treated using intravesical BCG.

## 2. Materials and Methods

### 2.1. Data Collection

This retrospective multicenter study was approved by the institutional review board of each participating institute (reference protocol ID: NMU-2217) of the Japan Urological Oncology Group framework [2]. Informed consent was obtained from participants through posters and/or website using the opt-out method [22]. We reviewed 3226 patients who received intravesical BCG for pathologically diagnosed NMIBC and treatment during 2000–2019 at 31 collaborative hospitals. The clinicopathological data included oncological outcomes, treatments, age, sex, performance status, history of NMIBC, tumor multiplicity, tumor size, T category, tumor grade (per 2004 WHO classification), and presence of bladder CIS, prostate-involving CIS, LVI, DD, and VMs.

### 2.2. Intravesical BCG Treatment after TURBT

The intravesical BCG schedule included weekly instillations of Immunobladder (Tokyo-172 strain) or ImmuCyst (Connaught strain, currently unavailable) for 6–8 consecutive weeks for induction BCG (iBCG) with or without subsequent maintenance BCG (mBCG). Planned maintenance protocol was BCG doses once a week for 3 weeks at 3, 6, 12, 18, 24, 30, and 36 months after iBCG initiation [23]. Adequate BCG’ therapy is when a patient has received at least five of six doses in induction phase followed by at least one maintenance (two of three dose) for clinical trials [24]. Based on the definition of adequate BCG, at least two of three doses in the first mBCG round at 3 months was considered mBCG implementation in this study.

### 2.3. Patient Selection

**Figure 1:**
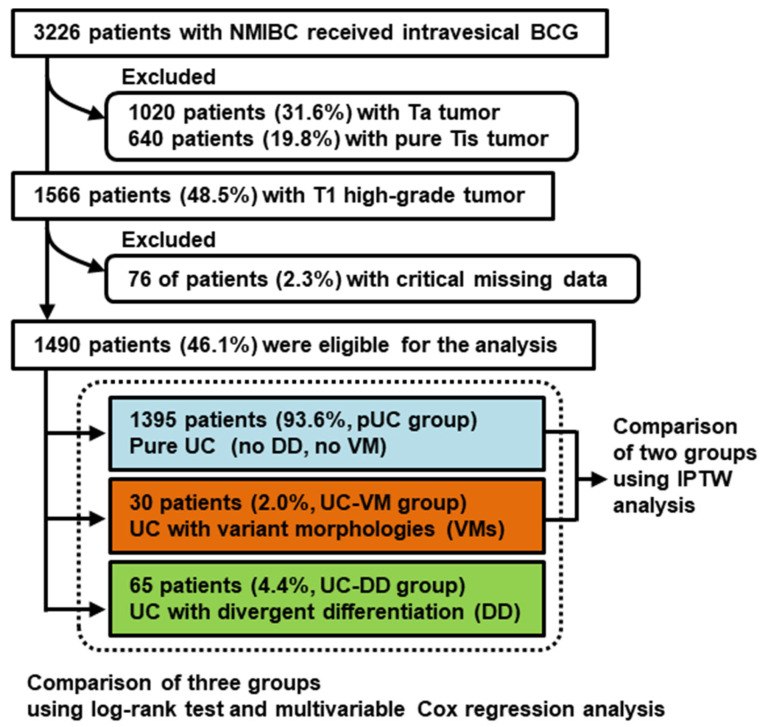
Flow chart for creation of the patient cohort dataset. From the original dataset, the cohort was restricted to T1 high-grade urothelial carcinoma (UC). Abbreviations: NMIBC, non-muscle invasive bladder cancer; BCG, Bacillus Calmette–Guérin; pUC, pure urothelial carcinoma; DD, divergent differentiation; VM, variant morphology; IPTW, inverse probability of treatment weighting.

Figure 1 shows a flowchart of the patient selection process. Of 3226 patients, the cohort was first restricted to 1490 (46.1%) including only T1 HG tumors. Next, 76 patients (2.3%) with critical missing data were excluded, leaving 1490 patients (46.1%) eligible for the analysis. Among 1490 patients, 1395 (93.6%) had pure UC (pUC group), 65 (4.4%) had UC with DD (UC-DD group), and only 30 (2.0%) had UC with VMs (UC-VM group).

### 2.4. Surveillance after Intravesical BCG and During mBCG

Although the surveillance protocol varied across institutions, patients were generally followed up using white-light cystoscopy and urinary cytology every 3 months for 2 years, then every 6 months in the third and fourth years, and annually thereafter [6]. Recurrence was defined as the presence of recurrent tumors of pathologically proven UC in the bladder and prostatic urethra. Neither recurrence of upper urinary tract nor positive result of urinary cytology without pathologically proven UC was considered recurrence. Progression was defined as recurrent disease with invasion into the muscularis propria (≥T2), positive regional lymph nodes, and/or distant metastases.

### 2.5. Statistical Analysis

Statistical analyses and were performed and plots were generated using GraphPad Prism version 7.00 (GraphPad Software, San Diego, CA, USA). All reported *p* values are two-sided, and statistical significance was set at *p* < 0.05. Clinicopathological characteristics were compared using Kruskal–Wallis tests or chi-square tests, as appropriate. Intravesical recurrence-free survival (RFS), progression-free survival (PFS), and cancer-specific survival (CSS) were calculated from the date of administration of the initial iBCG dose. Survival rates were analyzed using the Kaplan–Meier method and compared using the log-rank test among the pUC, UC-DD, and UC-VM groups. Variables that potentially affected prognosis (*p* < 0.1) in univariate analysis were included in a stepwise Cox proportional hazards regression model. Hazard ratio (HR) with 95% confidence interval (CI) was calculated to identify independent prognostic variables.

To minimize selection bias, inverse probability of treatment weighting (IPTW) analysis was performed using R version 4.0.0 (R Development Core Team, Vienna, Austria). IPTW, which is a form of propensity score analysis, uses weighting by the inverse of the propensity score to reduce imbalance in possible confounders between the pUC and UC-VM groups [25]. The baseline characteristics were matched between the pUC and UC-VM groups by calculating the propensity score for each patient using a multivariable logistic regression model based on covariates such as age, sex, multiplicity, tumor size, presence of concomitant bladder CIS, prostate-involving CIS, LVI on TUR specimen, implementation of second TUR, and implementation of mBCG. Differences in means and proportions between the two groups was quantified using standardized mean difference. Standardized mean difference more than 0.20 was considered to indicate potentially relevant imbalances between the two groups [25]. Multivariable Cox regression analysis was used to estimate the IPTW-adjusted HRs and 95% CIs as outcomes of the two groups.

## 3. Results

### 3.1. DD and VMs Detected in ur Cohort

**Figure 2:**
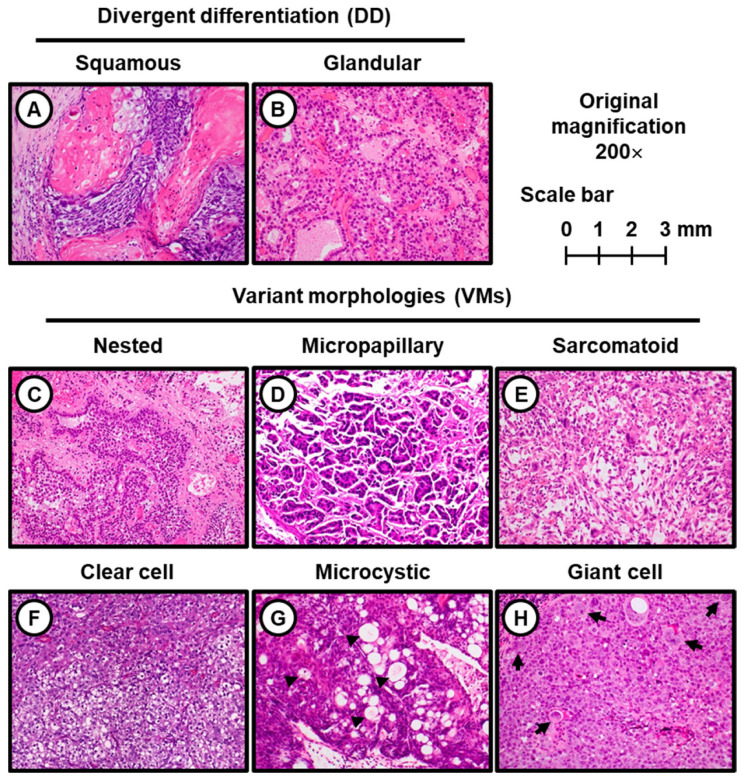
Divergent differentiation and variant morphologies in T1 high-grade urothelial carcinoma of cases from this study. All the illustrative images of hematoxylin-eosin staining were captured at original magnification 200×. (**A**) Squamous differentiation; (**B**) Glandular differentiation. This tumor has enteric features and mucin production; (**C**) Nested variant; (**D**) Micropapillary variant; (**E**) Sarcomatoid variant; (**F**) Clear cell variant; (**G**) Microcystic variant. This tumor forms neoplastic cystic structure. The lumina tend to be empty, but some of them contain necrotic cells, granular eosinophilic debris, or mucin (arrowheads); (**H**) Giant cell variant. This tumor has pleomorphic giant cells (black arrows) with cytoplasmic vacuoles.

Table 1 lists the distribution of unusual histology findings in the UC-DD and UC-VM groups. In the UC-DD group, 38 (69%) and 27 (41%) patients had glandular differentiation and squamous differentiation, respectively, whereas trophoblastic differentiation was not seen in our cohort. The most common VM was micropapillary variant (13/30; 43% in the UC-VM group). The second most common VM was nested variant (9/30, 30%). The third, sarcomatoid variant, was observed in 4 of 30 patients (13%), followed by clear cell variant (2/30, 6.7%), microcytic variant (1/30, 3.3%), and giant cell variant (1/30, 3.3%). Other VMs such as lymphoepithelioma-like, plasmacytoid, poorly differentiated, and lipid-rich variants were not present in our cohort. Representative hematoxylin & eosin-stained images of DD and VMs are shown in Figure 2.

### 3.2. Comparison of Patient Characteristics and Outcomes among the Groups

**Figure 3:**
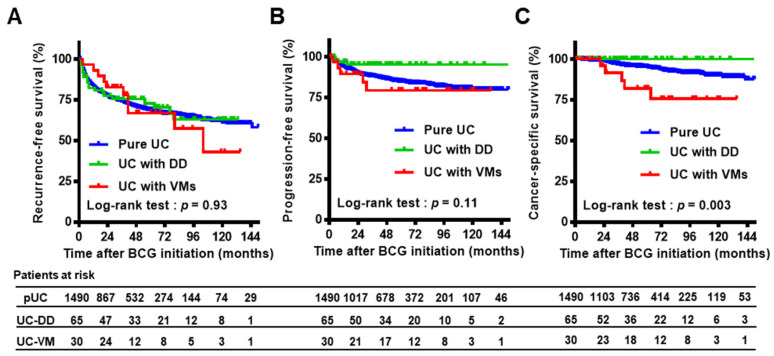
Survival curves of outcomes after initiation of intravesical BCG treatment among pure UC, UC with DD, and UC-VMs. Bladder recurrence-free survival (**A**), progression-free survival (**B**), and cancer-specific survival (**C**) were plotted and compared among three groups. Abbreviations: BCG, Bacillus Calmette–Guérin; UC, urothelial carcinoma; DD, divergent differentiation; VM, variant morphology.

Table S1 summarizes the patient characteristics of the three groups. Statistical comparisons showed significant differences in age, LVI, and implementation of second TUR. The positivity rate of LVI in TUR specimens and rate of second TUR implementation was higher in patients in the UC-VM group than in the pUC and UC-DD groups. Both the European Organization for Research and Treatment of Cancer (EORTC) [26] and the Spanish Urological Club for Oncological Treatment (CUETO) [27] risk tables for recurrence and progression incorporated six parameters to categorize patients into risk groups according to the summed scores. These score models were applied to our cohort to calculate each patient’s risk after intravesical BCG treatment, demonstrating that no significant difference was observed among each group (Table S1). In this cohort, 241 (16%) of 1490 patients received maintenance BCG and the median number of BCG doses in maintenance phase was 6 times (interquartile range, 3 to 9) and the mean number was 7.9 times. Of the 1490 patients, 466 (31%) and 199 (13%) experienced bladder cancer recurrence and progression, respectively, and 74 (5.0%) died of bladder cancer, with a median follow-up of 50 months (interquartile range, 27−79) after BCG initiation. We compared the clinical outcomes after induction of iBCG between Tokyo-172 strain (1141 patients) and Connaught strain (349 patients), demonstrating that no significant difference was observed for any of three endpoints. To investigate the impact of DD and VMs on oncological outcomes, RFS, PFS, and CSS were compared among the three groups using the Kaplan–Meier method and log-rank test (Figure 3 and Table S2). No significant impact of concomitant DD and VMs was seen for bladder recurrence. Compared with the pUC group, the UC-DD and UC-VM groups were associated with favorable prognosis and poor prognosis for progression (*p* = 0.08) and cancer-specific death (*p* < 0.01), respectively. Multivariate analysis revealed that concomitant VMs, not DD, was a strong independent factor for cancer-specific death (HR, 3.89; 95% CI, 1.55−9.77).

The UC-DD group displayed better survival outcomes than the pUC group (Figure 3). Based on this finding, we decided to further explore the clinical impact of VMs in terms of response to intravesical BCG. The background and outcomes of 30 patients in the UC-VM group are listed in Table S3. Of them, eight (27%) and five (17%) patients experienced bladder recurrence and progression, respectively, and five of the latter five patients experiencing progression died of bladder cancer. Notably, unresectable metastatic lesions occurred suddenly in three (no. 8, 15, and 24) of these five patients.

### 3.3. IPTW-Adjusted Comparison of Outcomes between the pUC and UC-VM Groups

IPTW analysis was applied to adjust for patient characteristics between the pUC and UC-VM groups and decrease the influence of possible confounding factors (Table 2). All weighted baseline characteristics included in the propensity score model were closely balanced between the two groups. Univariate and multivariate Cox regression analyses for RFS, PFS, and CSS with unadjusted cohort and the IPTW-adjusted model are shown in Tables S4–S6. Cox regression analysis using IPTW adjustment demonstrated that the impact of concomitant VMs was significant for CSS (Table 3; multivariate analysis; HR, 3.38; 95% CI, 1.92–5.93; *p* < 0.01) but not RFS (univariate analysis; HR, 0.86; 95% CI, 0.25–2.95; *p* = 0.81) and PFS (univariate analysis; HR, 1.88; 95% CI, 0.49–7.21; *p* = 0.36).

## 4. Discussion

We investigated the impact of concomitant DD and VMs in TUR specimens in T1/HG NMIBC patients who received intravesical BCG treatment. In our cohort, the frequency of some forms of DD or VMs was 6.4% (95/1490), which seemed to be much lower than that in previous studies [6,7,9,10]. This would be strongly attributed to biased patient selection. As our cohort included only T1/HG NMIBC patients treated with intravesical BCG treatment, we counted neither patients with NMIBC undergoing immediate RC nor MIBC patients. The European Association of Urology states that micropapillary, plasmacytoid, and sarcomatoid variants are associated with poorer prognosis [3]. In addition, the National Comprehensive Cancer Network Clinical guidelines recommend immediate RC for T1 NMIBC with micropapillary, plasmacytoid, and sarcomatoid variants [4]. However, this real-world data displayed treatment outcomes of a few cases with rare VMs, such as sarcomatoid, clear cell, microcytic, and giant cell variants. Although there were no patients with the plasmacytoid variant here, our findings highlight the favorable response to intravesical BCG against T1 disease with rare VMs.

Metastatic lesion occurs usually after local progression to MIBC in patients with T1 bladder UC. In our cohort, unresectable metastatic lesions occurred suddenly in three (60%) of five UC-VMs patients who experienced disease progression. Concomitant VMs can be characterized by a highly metastatic potential. As to micropapillary variant, a large multi-institutional cohort study demonstrated that micropapillary variant was associated with higher pathologic stage and LVI in MIBC patients who underwent radical cystectomy [28]. This finding strongly implies UC with micropapillary variant presents higher capability of invasion and metastasis. Regarding the prognosis of T1 UC with micropapillary variant, previous study by Willis et al. [19] concluded improved survival was seen in those patients who underwent immediate RC, while some patients may respond to intravesical BCG. However, future precision medicine such as detailed molecular analysis would be required to identify subsets who can be managed by bladder-preservation therapy. To date, various types of analyses have been reported to evaluate the efficacy of intravesical BCG for NMIBC with DD or VMs. Yorozuya et al. [29] compared the outcomes between BCG-treated and non-BCG-treated patients with UC with DD, concluding that intravesical BCG may provide clinical benefit. Suh et al. conducted comparative analysis of immediate RC, intravesical BCG, and no treatment groups for UC with DD, demonstrating that intravesical BCG could be an appropriate treatment option [30]. Furthermore, Shapur and Gofrit et al. reported unfavorable outcomes of 22 NMIBC patients with concomitant DD or VMs treated with intravesical BCG [21]. The same group later compared the outcomes after intravesical BCG between 41 patients with NMIBC with DD or VMs and 140 control patients with pure UC, interpreting that concomitant DD or VMs was associated with significantly worse prognosis [20]. However, these retrospective studies did not separately evaluate the UC-DD and UC-VM groups, and thus, do not provide insight into their differences. Our study adopted a two-step prognostic analysis: conventional multivariate Cox regression analysis for all three groups (pUC, UC-DD, and UC-VM) and subsequent IPTW-adjusted analysis for the selected two groups (pUC and UC-VM). Our first-step analysis revealed favorable survival outcomes of UC-DD groups that were consistent with the results from previous studies [29,30]. Notably, Gofrit et al. [20] reported that none of nine patients with glandular differentiation experienced bladder recurrence, progression, or cancer-specific death. Similarly, no patient with glandular or squamous differentiation in our cohort died of bladder cancer progression (Figure 3C).

Another issue to be discussed is underdiagnosis and misdiagnosis of rare histological UC variants in pathological reports for TUR specimens. A previous study demonstrated that the involvement of experienced uropathologists in the process of pathological diagnosis is of great importance, increasing the detection rate of UC variants, especially for sarcomatoid variants [31]. Regarding the concordance between histological diagnoses for TUR and RC specimens, the detection rate of UC variants was 6.4% and 14.1% of TUR and RC specimens, respectively [32]. Despite significant lack of concordance in UC variants between TUR and RC specimens, concomitant DDs or VMs were still associated with unfavorable clinical outcomes. Unfortunately, the involvement of experienced uropathologists did not increase the concordance rate between histological diagnoses of UC variants for TUR and RC specimens [31], suggesting that underdiagnosis of UC variants at TUR specimens might be unavoidable even with the involvement of experienced uropathologists in the diagnostic process.

This study had several limitations. First, its retrospective nature had an inherent potential for selection bias; for example, the criteria, dose, and schedule of BCG treatment depended on the institutional protocol and physician’s discretion. The cohort was derived from multiple institutions, which could introduce inconsistencies in surgical skills, clinical interpretations, and pathological diagnoses. Second, because all the collaborative institutes are academic hospitals, it was assumed that the pathologists were well-experienced with urogenital cancer diagnosis. However, the pathological skill and insight could vary among institutes. Third, we did not include patients who underwent immediate RC and did not evaluate how beneficial intravesical BCG was when compared with the RC-treated or the untreated group. The rate of selection of immediate RC and other alternative treatment and the outcome of those patients are not available. Finally, we did not evaluate the possible impact of the amount of concomitant DD or VMs on the diagnosis. The 2016 WHO blue book recommends that pathologists report the percentage of variants in the pathology report.

## 5. Conclusions

Except for some rare variants, DD or VMs are often seen in bladder UC. Our two-step prognostic analysis clearly demonstrated that UC-VM was associated with poor outcomes in comparison to UC-DD. We conducted IPTW-adjusted analysis to investigate the real clinical impact of concomitant VMs. This aggressive subset should be clearly separated from UC-DD in terms of response to intravesical BCG. Our study should help both urologists and pathologists better understand the clinical and biological behavior of UC-VM and the potential of bladder-preservation therapy using intravesical BCG. We believe that our findings will help establish optimized treatment strategies such as precision medicine for this aggressive UC subset.

## Figures and Tables

**Table 1:** Distribution of divergent differentiation (DD) and variant morphologies (VMs) in this cohort.

Histology	UC with DD (UC-DD Group)	UC with VMs (UC-VM Group)
*N*	65 (100%)	30 (100%)
Glandular differentiation	38 (69%)	-
Squamous differentiation	27 (41%)	-
Micropapillary variant	-	13 (43%)
Nested variant	-	9 (30%)
Sarcomatoid variant	-	4 (13%)
Clear cell variant	-	2 (6.7%)
Microcystic variant	-	1 (3.3%)
Giant cell variant	-	1 (3.3%)

Abbreviations: UC, urothelial carcinoma.

**Table 2:** Baseline characteristics of patients with T1 high-grade tumor who received intravesical Bacillus Calmette–Guérin (BCG) treatment—before and after adjustment.

Valiables		Unweighted Population (n)				IPTW Population		
		Pure UC (pUC)	UC with VMs (UC-VM)	*p* Value	SMD	Pure UC (pUC)	UC with VMs (UC-VM)	SMD
*N*		1395	30			1425	1442	
Age, mean ± SD		70.7 ± 9.5	67.4 ± 9.8	0.06 #	0.35	70.7 ± 9.4	69.8 ± 1.4	0.13
Sex				1.00 ##	0.014			0.16
	Male	1155 (83%)	25 (83%)			83%	88%	
	Female	240 (17%)	5 (17%)			17%	12%	
Multiplicity				0.13 ##	0.28			0.19
	Single	503 (36%)	15 (50%)			36%	27%	
	Multiple	892 (64%)	15 (50%)			64%	73%	
Tumor size				0.83 ##	0.021			0.047
	<3 cm	1082 (78%)	23 (77%)			77%	79%	
	≥3 cm	313 (22%)	7 (23%)			23%	21%	
Bladder CIS				0.85 ##	0.044			0.132
	No	854 (61%)	19 (63%)			61%	45%	
	Yes	541 (39%)	11 (37%)			39%	55%	
Prostate-involving CIS				1.00 ##	0.199			0.197
	No	1368 (98%)	30 (100%)			98%	100%	
	Yes	27 (1.9%)	0 (0%)			1.9%	0%	
LVI				<0.01 ##	0.64			0.030
	No	1275 (91%)	20 (67%)			91%	92%	
	Yes	120 (9.1%)	10 (33%)			9.1%	8.3%	
Second TUR				0.14 ##	0.32			0.026
No		629 (45%)	9 (30%)			45%	46%	
	Yes	766 (55%)	21 (70%)			55%	54%	
Maintenance BCG				0.81 ##	0.085			0.20
	No	1167 (84%)	26 (87%)			84%	76%	
	Yes	228 (16%)	4 (13%)			16%	24%	

NMIBC, non-muscle invasive bladder cancer; IPTW, inverse probability of treatment weighting; UC, urothelial carcinoma; VMs, variant morphologies; SMD, standardized mean difference; SD, standard deviation; CIS, carcinoma in situ; LVI, lymphovascular invasion; TUR, transurethral resection; # Kruskal-Wallis test; ## Chi-square test.

**Table 3:** Inverse probability of treatment weighting (IPTW)-adjusted multivariate Cox proportional hazard regression analysis for oncological outcomes.

Oncological Outcomes	Variables		HR	95% CI	*p* Value
Bladder Recurrence-Free Survival					
	Age	≥70 yo/<70 yo	1.31	1.07–1.60	<0.01
	Multiplicity	multiple/solitary	1.46	1.18–1.80	<0.01
	Prostate-involving CIS	yes/no	2.96	1.82–4.79	<0.01
	LVI	yes/no	1.45	1.06–1.97	0.02
	Second TUR	yes/no	0.73	0.60–0.89	<0.01
	Maintenance BCG	yes/no	0.49	0.36–0.68	<0.01
Progression-free survival					
	Age	≥ 70 yo/<70 yo	1.25	0.93–1.67	0.14
	Prostate-involving CIS	yes/no	3.38	1.92–5.93	<0.01
	LVI	yes/no	1.56	1.02–2.37	0.04
	Second TUR	yes/no	0.75	0.56–1.00	0.05
	Maintenance BCG	yes/no	0.59	0.37–0.94	0.03
Cancer-specific survival					
	Age	≥ 70 yo/<70 yo	1.90	1.15–3.13	0.01
	Histology type	UC-VM/pUC	3.38	1.92–5.93	<0.01
	Second TUR	yes/no	0.58	0.34–0.97	0.04

HR, hazard ratio; CI, confidence interval; CIS, carcinoma in situ; LVI, lymphovascular invasion; TUR, transurethral resection; BCG, Bacillus Calmette-Guérin; UC-VM, urothelial carcinoma with variant morphologies; pUC, pure urothelial carcinoma.

## Data Availability

The data presented in this study are available in present article and supplementary.

## Supplementary Materials

The following are available online at https://www.mdpi.com/article/10.3390/cancers13112615/s1, Table S1: Clinicopathological variables of patients with T1 high-grade tumor treated with intravesical Bacillus Calmette–Guérin (BCG) and comparison according to divergent differentiation (DD) and variant morphologies (VM)., Table S2: Prognostic variables in patients with T1 high-grade tumor treated using intravesical Bacillus Calmette–Guérin (BCG)., Table S3: Background and outcomes of 30 patients with T1 high-grade tumor with variant morphologies (VMs). Table S4: Cox proportional hazard regression models for bladder recurrence-free survival., Table S5: Cox proportional hazard regression models for progression-free survival., Table S6: Cox proportional hazard regression models for cancer-specific survival.
