# Supplementary material for: Intravesical Bacillus Calmette–Guérin Treatment for T1 High-Grade Non-Muscle Invasive Bladder Cancer with Divergent Differentiation or Variant Morphologies
Source: Cancers (Basel). 2021 May 26;13(11):2615. doi: 10.3390/cancers13112615 (PMC8198171; doi:10.3390/cancers13112615)
Supplement: Supplementary file 1 [file cancers-13-02615-s001.zip › cancers-1212971-supplementary.pdf]

cancers

MDPI

Supplementary

Intravesical Bacillus Calmette–Guérin Treatment for t1 High-Grade Non-Muscle Invasive Bladder Cancer with Divergent Differentiation or Variant Morphologies

Makito Miyake et. al.

Table 1. Clinicopathological variables of patients with T1 high-grade tumor treated with intravesical Bacillus Calmette–Guérin (BCG) and comparison according to divergent differentiation (DD) and variant morphologies (VM).

Variables	Total	Histological Type			Comparison of Three Groups
		Pure UC (pUC group)	UC with DD (UC-DD group)	UC with VMs (UC-VM group)	
N	1490 (100%)	1395 (94%)	65 (4.4%)	30 (2.0%)	-
Age, mean ± SD	70.7 ± 9.5	70.7 ± 9.5	70.8 ± 10.1	67.4 ± 9.8	0.01 #
Sex					0.09 ##
Male	1227 (82%)	1155 (83%)	47 (72%)	25 (83%)	
Female	263 (18%)	240 (17%)	18 (28%)	5 (17%)	
Multiplicity					0.27 ##
Single	543 (36%)	503 (36%)	25 (39%)	15 (50%)	
Multiple	947 (64%)	892 (64%)	40 (61%)	15 (50%)	
Tumor size					0.10 ##
<3 cm	1148 (77%)	1082 (78%)	43 (66%)	23 (77%)	
^{3} 3 cm	342 (23%)	313 (22%)	22 (34%)	7 (23%)	
Bladder CIS					0.76 ##
No	910 (61%)	854 (61%)	37 (57%)	19 (63%)	
Yes	580 (39%)	541 (39%)	28 (43%)	11 (37%)	
Prostate-involving CIS					0.72 ##
No	1462 (98%)	1368 (98%)	64 (98%)	60 (100%)	
Yes	28 (1.9%)	7 (1.9%)	1 (1.5%)	0 (0%)	
LVI					<0.0001 ##
No	1355 (91%)	1275 (91%)	60 (92%)	20 (67%)	

Yes	135 (9.1%)	120 (8.6%)	5 (7.7%)	7 (33%)	
EORTC recurrence score †, mean ± SD	6.1 ± 2.2	6.1 ± 2.2	6.3 ± 2.2	5.7 ± 2.5	0.25 #
EORTC progression score †, mean ± SD	12.9 ± 4.4	12.9 ± 4.4	13.6 ± 4.5	12.9 ± 4.2	0.45 #
CUETO recurrence score ‡, mean ± SD	5.8 ± 2.5	5.8 ± 2.5	6.2 ± 3.1	5.3 ± 2.0	0.40 #
CUETO progression score ‡, mean ± SD	8.6 ± 2.2	8.5 ± 2.2	8.8 ± 2.5	8.6 ± 1.5	0.71 #
Second TUR					<0.0001 ##
No	649 (44%)	629 (45%)	11 (17%)	9 (30%)	
Yes	841 (56%)	766 (55%)	54 (83%)	21 (70%)	
Maintenance BCG					0.79 ##
No	1249 (84%)	1167 (84%)	56 (86%)	26 (87%)	
Yes	241 (16%)	228 (16%)	9 (14%)	4 (13%)	

Abbreviations: BCG, Bacillus Calmette–Guérin; DD, divergent differentiation; VMs, variant morphologies; SD, standard deviation; CIS, carcinoma in situ; LVI, lymphovascular invasion; EORTC, the European Organisation for Research and Treatment of Cancer; CUETO, the the Spanish Urological Club for Oncological Treatment; TUR, transurethral resection; # Kruskal-Wallis test; ## Chi-square test; † Scores are calculated based on the citation no. 25 (Sylvester RJ et al, 2006).; ‡ Sores are calculated based on the citation no. 26 (Fernandez-Gomez J et al, 2009).

Table S2. Prognostic variables in patients with T1 high-grade tumor treated using intravesical Bacillus Calmette–Guérin (BCG).

Variables	Bladder recurrence-free survival						Progression-free survival						Cancer-specific survival					
	Univariate			Multivariate			Univariate			Multivariate			Univariate			Multivariate		
	HR	95% CI	*p* Value	HR	95% CI	*p*-Value	HR	95% CI	*p* Value	HR	95% CI	*p* Value	HR	95% CI	*p* value	HR	95% CI	*p* Value
Age																		
<70	1			1			1			1			1			1		
≥70	1.36	1.11–1.65	<0.01	1.31	1.07–1.60	< 0.01	1.29	0.97–1.71	0.08	1.25	0.94–1.67	0.12	2.23	1.40–3.54	< 0.01	1.89	1.17–3.07	<0.01
Sex																		
Male	1						1						1					
Female	1.00	0.78–1.28	0.98				0.86	0.59–1.26	0.43				0.88	0.50–1.56	0.66			
Multiplicity																		
Single	1			1			1						1					
Multiple	1.49	1.21–1.84	< 0.01	1.45	1.18–1.80	< 0.01	1.22	0.91–1.65	0.18				1.43	0.90–2.29	0.13			
Tumor size																		
<3 cm	1						1						1					
≥3 cm	1.11	0.89–1.39	0.34				1.07	0.78–1.48	0.67				1.23	0.76–2.00	0.40			
Bladder CIS																		

No	1						1						1					
Yes	1.05	0.86–1.28	0.63				1.13	0.85–1.50	0.39				0.75	0.47–1.20	0.23			
Prostate-involving CIS																		
No	1			1			1			1			1					
Yes	3.41	2.13–5.48	< 0.01	2.99	1.84–4.87	< 0.01	4.17	2.37–7.31	< 0.01	3.72	2.11–6.55	< 0.01	1.97	0.62–6.23	0.25			
LVI																		
No	1			1			1			1			1					
Yes	1.52	1.13–2.04	< 0.01	1.47	1.09–1.98	0.01	1.69	1.13–2.54	0.01	1.59	1.05–2.39	0.03	1.16	0.56–2.41	0.69			
Histology type																		
Pure UC	1						1			1			1					
UC with DD	0.91	0.56–1.48	0.70				0.33	0.10–1.03	0.06	0.36	0.12–1.14	0.08	NA					
UC with VMs	0.78	0.37–1.65	0.51				1.24	0.51–3.02	0.63	1.24	0.51–3.05	0.64	3.38	1.36–8.38	< 0.01	3.89	1.55–9.77	< 0.01
Second TUR																		
No	1			1			1			1			1			1		
Yes	0.72	0.60–0.88	< 0.01	0.73	0.60–0.89	< 0.01	0.74	0.56–0.98	0.04	0.77	0.58–1.03	0.07	0.56	0.36–0.88	0.01	0.60	0.37–0.97	<0.01
Maintenance BCG																		
No	1			1			1			1			1					
Yes	0.47	0.34–0.64	< 0.01	0.52	0.38–0.72	< 0.01	0.57	0.36–0.89	0.01	0.62	0.39–0.98	0.04	0.95	0.52–1.71	0.85			

Abbreviations: NMIBC, non-muscle invasive bladder cancer; BCG, Bacillus Calmette–Guérin; HR, hazard ratio; CI, confidence interval; CIS, carcinoma in situ; LVI, lymphovascular invasion; TUR, transurethral resection; UC,urothelial carcinoma; DD, divergent differentiation; VMs, variant morphologies; NA, not available because no events seen in this disease subset.

Table S3. Background and outcomes of 30 patients with T1 high-grade tumor with variant morphologies (VMs).

Patient No.	Age	Sex	VMs	CIS	LVI	Bladder recurrence	Progression	Pattern of progression	Survival outcome
1	73	M	Micropapillary	+		No	No		Alive, NED (81 months)
2	70	M	Micropapillary	+		No	No		Alive, NED (60 months)
3	48	M	Micropapillary	+		No	No		Alive, NED (52 months)
4	60	M	Micropapillary	+	+	No	No		Alive, NED (98 months)
5	69	M	Micropapillary		+	No	No		Alive, NED (21 months)
6	81	M	Micropapillary	+	+	Yes (4 months)	No		Dead of other cancer (20 months)
7	66	F	Micropapillary			No	No		Alive, NED (61 months)
8	61	M	Micropapillary			Yes (9 months)	Yes (9 months)	to MIBC and LN metastasis	Dead of BCa (41 months)
9	69	M	Micropapillary	+		No	No		Alive, NED (6 months)
10	54	M	Micropapillary	+		No	No		Alive, NED (119 months)

11	70	M	Micropapillary			No	No		Alive, NED (121 months)
12	62	M	Micropapillary			No	No		Alive, NED (78 months)
13	57	M	Micropapillary		+	No	No		Dead of other cancer (41 months)
14	72	M	Nested		+	Yes (7 months)	Yes (7 months)	to MIBC (T2)	Dead of BCa (21 months)
15	85	M	Nested	+		Yes (3 months)	Yes (3 months)	to MIBC and Adrenal metastasis	Dead of Bca (25 months)
16	60	M	Nested			No	No		Dead of other cancer (135 months)
17	66	M	Nested	+		No	No		Alive, NED (10 months)
18	70	M	Nested		+	Yes (31 months)	Yes (31 months)	to MIBC	Dead of BCa (63 months)
19	76	F	Nested		+	Yes (57 months)	0		Alive, NED (80 months)
20	73	F	Nested	+		No	No		Alive, NED (100 months)
21	73	M	Nested			Yes (6 months)	No		Alive, NED (74 months)
22	53	M	Nested			No	No		Alive, NED (72 months)
23	63	M	Sarcomatoid		+	No	No		Alive, NED (23 months)
24	62	M	Sarcomatoid			Yes (4 months)	Yes (28 months)	Bone metastasis	Dead of BCa (39 months)
25	53	M	Sarcomatoid	+		No	No		Alive, NED (58 months)
26	88	M	Sarcomatoid			No	No		Alive, NED (1 months)
27	70	M	Clear cell			No	No		Alive, NED (101 months)
28	71	F	Clear cell			No	No		Alive, NED (110 months)
29	62	M	Microcytic		+	No	No		Alive, NED (14 months)
30	85	F	Giant cell		+	No	No		Dead of unknown cause (135 months)

Abbreviations: UC, urothelial carcinoma; VMs, variant morphologies; CIS, carcinoma in situ; LVI, lymphovascular invasion; MIBC, muscle invasive bladder cancer; LN, lymph node; NED, no evidence of disease; BCa, bladder cancer.

Table S4. Cox proportional hazard regression models for bladder recurrence-free survival.

Variables	Univariate			Multivariate			Univariate (IPTW Adjusted)			Multivariate (IPTW Adjusted)		
	HR	95% CI	*p* value	HR	95% CI	*p* value	HR	95% CI	*p* value	HR	95% CI	*p* value
Age												
<70	1			1			1			1		
≥70	1.38	1.13–1.69	<0.01	1.02	1.01–1.03	<0.01	1.38	1.13–1.69	< 0.01	1.31	1.07–1.60	< 0.01
Sex												
Male	1						1					
Female	0.97	0.75–1.26	0.84				0.97	0.75–1.26	0.81			
Multiplicity												
Single	1			1			1			1		

Multiple	1.44	1.16–1.78	< 0.01	1.43	1.16–1.77	< 0.01	1.44	1.17–1.78	< 0.01	1.46	1.18–1.80	< 0.01
Tumor size												
<3 cm	1						1					
≥3 cm	1.12	0.89–1.40	0.35				1.10	0.87–1.39	0.42			
Bladder CIS												
No	1						1					
Yes	1.03	0.84–1.26	0.79				1.04	0.85–1.27	0.72			
Prostate-involving CIS												
No	1			1			1			1		
Yes	3.31	2.03–5.38	< 0.01	3.02	1.86–4.92	< 0.01	3.29	1.98–5.46	<0.01	2.96	1.82–4.79	<0.01
LVI												
No	1			1			1			1		
Yes	1.52	1.13–2.04	<0.01	1.43	1.06–1.92	0.02	1.49	1.10–2.04	0.01	1.45	1.06–1.97	0.02
Histology type												
Pure UC	1						1					
UC with VMs	0.78	0.37–1.65	0.51				0.86	0.25–2.95	0.81			
Second TUR												
No	1			1			1			1		
Yes	0.71	0.59–0.87	< 0.01	0.74	0.61–0.90	< 0.01	0.72	0.59–0.87	< 0.01	0.73	0.60–0.89	< 0.01
Maintenance BCG												
No	1			1			1			1		
Yes	0.48	0.35–0.67	< 0.01	0.53	0.38–0.74	< 0.01	0.46	0.34–0.64	< 0.01	0.49	0.36–0.68	< 0.01

Abbreviations: HR, hazard ratio; CI, confidence interval; IPTW, inverse probability of treatment weighting; CIS, carcinoma in situ; LVI, lymphovascular invasion; UC,urothelial carcinoma; VMs, variant morphologies; TUR, transurethral resection; BCG, Bacillus Calmette-Guérin.

Table 5. Cox proportional hazard regression models for progression-free survival.

Variables	Univariate			Multivariate			Univariate (IPTW adjusted)			Multivariate (IPTW adjusted)		
	HR	95% CI	*p* Value	HR	95% CI	*p* Value	HR	95% CI	*p* Value	HR	95% CI	*p* Value
Age												
<70	1			1			1			1		
≥70	1.28	0.96–1.71	0.09	1.25	0.94–1.67	0.13	1.28	0.96–1.72	0.09	1.25	0.93–1.67	0.14
Sex												
Male	1						1					
Female	0.90	0.62–1.32	0.59				0.89	0.61–1.30	0.56			

Multiplicity												
Single	1						1					
Multiple	1.18	0.88–1.59	0.27				1.2	0.88–1.62	0.25			
Tumor size												
<3 cm	1						1					
≥3 cm	1.07	0.77–1.48	0.70				1.03	0.74–1.44	0.87			
Bladder CIS												
No	1						1					
Yes	1.12	0.84–1.49	0.44				1.17	0.87–1.57	0.29			
Prostate-involving CIS												
No	1			1			1			1		
Yes	3.76	2.09–6.74	< 0.01	3.42	1.90–6.15	< 0.01	3.69	2.14–6.38	< 0.01	3.38	1.92–5.93	< 0.01
LVI												
No	1			1			1			1		
Yes	1.70	1.13–2.55	0.01	1.56	1.03–2.36	0.03	1.67	1.10–2.53	0.02	1.56	1.02–2.37	0.04
Histology type												
Pure UC	1						1					
UC with VMs	1.24	0.51–3.02	0.63				1.88	0.49–7.21	0.36			
Second TUR												
No	1			1			1			1		
Yes	0.75	0.57–1.00	0.05	0.77	0.58–1.03	0.08	0.73	0.55–0.98	0.03	0.75	0.56–1.00	0.05
Maintenance BCG												
No	1			1			1			1		
Yes	0.57	0.36–0.89	0.01	0.63	0.40–0.99	0.05	0.55	0.35–0.87	< 0.01	0.59	0.37–0.94	0.03

Abbreviations: HR, hazard ratio; CI, confidence interval; IPTW, inverse probability of treatment weighting; CIS, carcinoma in situ; LVI, lymphovascular invasion; UC, urothelial carcinoma; VMs, variant morphologies; TUR, transurethral resection; BCG, Bacillus Calmette-Guérin.

Table S6. Cox proportional hazard regression models for cancer-specific survival.

Variables	Univariate			Multivariate			Univariate (IPTW Adjusted)			Multivariate (IPTW Adjusted)		
	HR	95% CI	*p* Value	HR	95% CI	*p* Value	HR	95% CI	*p* Value	HR	95% CI	*p* Value
Age												
<70	1			1			1			1		
≥70	1.94	1.20–3.16	< 0.01	1.89	1.17–3.07	< 0.01	2.26	1.40–3.65	< 0.01	1.90	1.15–3.13	0.01
Sex												

Male	1						1					
Female	0.78	0.41–1.48	0.45				0.77	0.40–1.47	0.42			
Multiplicity												
Single	1						1					
Multiple	1.26	0.77–2.04	0.36				1.34	0.81–2.22	0.26			
Tumor size												
<3 cm	1						1					
≥3 cm	1.33	0.80–2.21	0.27				1.19	0.71–2.01	0.51			
Bladder CIS												
No	1						1					
Yes	0.77	0.47–1.26	0.29				0.90	0.52–1.56	0.72			
Prostate-involving CIS												
No	1						1					
Yes	2.41	0.76–7.67	0.14				2.32	0.72–7.52	0.16			
LVI												
No	1						1					
Yes	1.53	0.78–2.97	0.21				1.31	0.65–2.65	0.46			
Histology type												
Pure UC	1			1			1			1		
UC with VMs	3.38	1.36–8.38	< 0.01	3.89	1.55–9.77	< 0.01	4.67	1.25–17.45	0.02	4.76	1.25–18.23	0.02
Second TUR												
No	1			1			1			1		
Yes	0.62	0.39–0.99	0.05	0.6	0.37–0.97	0.04	0.57	0.35–0.95	0.03	0.58	0.34–0.97	0.04
Maintenance BCG												
No	1						1					
Yes	1.09	0.60–1.98	0.79				1.04	0.56–1.90	0.91			

Abbreviations: HR, hazard ratio; CI, confidence interval; IPTW, inverse probability of treatment weighting; CIS, carcinoma in situ; LVI, lymphovascular invasion; UC, urothelial carcinoma; VMs, variant morphologies; TUR, transurethral resection; BCG, Bacillus Calmette-Guérin.
